# Understanding merger control remedies in China

**DOI:** 10.1371/journal.pone.0336795

**Published:** 2025-11-21

**Authors:** Shengyan Hu, Jiong Gong

**Affiliations:** 1 School of Economics and Management, Huangshan University, Anhui, China; 2 School of International Trade and Economics, University of International Business and Economics, Beijing, China; Middle Tennessee State University Jennings A Jones College of Business, UNITED STATES OF AMERICA

## Abstract

We identified 8 merger cases with remedies in China for a period from the time when the Anti-monopoly Law was enacted in 2008–2018, and about 150 other merger cases corresponding to these 8 cases in their respective industries but approved without remedies. We then use the latter data to construct a counterfactual for the former to compare the factually observed level of competition after the merger decision with that derived from the counterfactual, based on a method called the synthetic control method (SCM). The exercise allows us to assess the remedies’ effectiveness. We find that overall the remedies are effective, but the structural remedies’ effect tends to be more abrupt and pronounced than behavioral remedies.

## 1. Introduction

After China’s accession to the WTO in 2001, the landscape of the market economy experienced significant structural changes, characterized by substantial inflows of multinational capital and an increase in domestic mergers and acquisitions among enterprises [1,2]. At the same time, the existing competition law and regulation system has revealed three critical institutional shortcomings: firstly, there was inadequate regulation of monopolistic agreements, particularly concerning vertical restrictions on competition; secondly, there was a complete lack of a review mechanism for the concentration of undertakings; and thirdly, there were insufficient constraints on administrative monopolies [3]. In view of these, there is an urgent necessity to establish a foundational legal framework for competition policy compatible with a modern market economy. Against this background, the Anti-Monopoly Law (AML) was officially enacted and implemented in 2008, marking the first instance in which the law articulated three primary objectives, as delineated in Article 1 of the AML: to “protect fair competition in the market” to “enhance the efficiency of economic operation” and to “safeguard consumer interests and the public interest.”

The purpose of a competition authority’s merger control process is to ensure the concentration of undertakings causing no adverse impact on market competition. Every year there are hundreds of merger and acquisition (M&A) cases filed with competition authorities for antitrust review in China. Since the implementation of the AML in August, 2008, the competition authority has issued 3 review decisions of rejection, 57 review decisions of conditional approval with remedies attached, and 4,760 review decisions of unconditional approval, as of December 31, 2022. Out of the 57 conditional approval cases, 10 have imposed structural remedies, such as the divestiture of assets or businesses; 36 with behavioral remedies, such as the licensing of key technologies (including patents, know-how and other intellectual property rights), termination of exclusionary agreements and etc., and 11 with hybrid remedies, by which we mean both structural and behavioral remedies are applied.

This paper attempts to study the effectiveness of the types of remedies in reducing or eliminating the anti-competitive effect of the concentration. While there is a sizable body of publications addressing the merger review effectiveness issue in the context of the U.S. and the European Union markets, systematic and quantitative studies of merger control remedies in China are lacking in the literature. In this paper, we find that remedies do generally play a positive role in preserving market competition, but to varying degrees depending on the types of remedies imposed. In general, structural and behavioral remedies consistently display a different effect on market competition over time, although eventually they tend to achieve more or less the same objective.

Our contribution to the existing literature is grounded in the application of a novel method inspired by the instrumental work of Carlton [4], who argues that retrospective studies of ex-post price change comparisons may be inadequate in that they typically do not incorporate the competition authority’s expectations of the post-merger market. In other words, the ex-post price comparisons may not be about a matter of increase or decrease in price, but rather a matter of increase or decrease over a level of the price change that the competition authority has already envisioned in mind and possibly tolerated as such due to other possible reasons related to market conditions.

Carlton’s argument is controversial, as the stated mission of most competition authorities is indeed to preserve competition in fact. However, it does raise a legitimate issue with respect to the exact price comparison benchmark, whether being the pre-merger level or some hypothetical level of a similarly-situated market that may have seen an increase in prices for various reasons but nevertheless the competition authority has possibly tolerated. In this paper, we advocate for the consideration of both benchmarks. In the context of studying merger remedies, the analogous issue in our case then should be about remedies being able to both preserve the existing level of competition post-merger, and any deviation from the level of competition had there been no remedies at all. In the latter case, a price increase could be the result of the competition authority’s possible tolerance or possibly its failure of regulatory oversight. This guiding philosophy essentially ordains an approach to construct a counterfactual without remedies, against which the observed level of post-merger competition can be evaluated. And this is exactly what we try to achieve in this paper.

We adopt a method called the synthetic control method proposed by Abadie and Gardeazabal [5], which has been extensively used in evaluating the impact of environmental changes, health, and policies [6–10]. In the area of merger control assessment, Gugler and Szücs [11] used the synthetic control method to evaluate 183 large mergers that underwent the scrutiny of European competition law between 1990 and 2007. In a book compiled by the Swedish Competition Authority, Mehta and Miller [12] recommended it as one of the methods for choosing the appropriate control group in evaluation. Generally the idea is to construct a counterfactual based on the data from a control group that bears a great deal of resemblance to the entity at issue, and then compare the counterfactual with the actually observed factual data. In our context, for every conditionally approved merger case with remedies, we select all the unconditionally approved merger cases of the same industry as a control group. We then use the data of the latter germane to competition to construct a counterfactual competition trajectory for the former, and then compare this counterfactual with the observed competition level to see: 1). if it exceeds or falls short of the synthetic pre-merger competition level of the control group that we construct out of the counterfactual; and 2). if it exceeds or falls short of the post-merger competition level of the synthetic construct. If the answer is yes in the first comparison, it leads to a definitive conclusion of regulatory oversight success. But the implication of the yes answer in the second comparison is a bit complicated, as it leads to two possibilities. One is that the competition authority succeeds in preserving a competition level that the competition authority has already envisioned in mind. Or it could be the case that the unconditional approvals in the control group were all wrong on average in the first place, such that even if the current case performs better than the control group, it might still fail to preserve competition as it should have.

Our choice of the variable to measure competition is the merger entity’s gross margin, which is calculated based on the sales revenue and the cost of goods sold obtained from income statements in publicly listed firms’ financial disclosures. Gross margin is also often used as a proxy for the classic price-cost margin metric to measure a firm’s market power, which essentially reflects the level of competition in the marketplace, if there is competition, as manifested in the Herfindahl–Hirschman index aggregated over the entire industry. Loecker, Eeckhout [13] used a similar markup metric to measure the rise in market power of the U.S. economy since 1980s. In a study of antitrust implications of global mergers over a period of 15 years, Gugler, Mueller [14] constructed a measure of profitability changes based on changes in the ratios of profits to total assets. What these papers have in common is to focus on changes in some measurement of profitability as a means to reflect changes in market power, and our paper follows the same line of thinking.

We concede that the majority of the large body of the ex-post antitrust assessment literature, i.e., the literature of empirically examining the effectiveness of merger control, focuses on changes in prices affected by the approval decision. Many of these studies use retail scanner data. In our case involving the counterfactual construction with a control group, it would be difficult to use pricing data in consideration of the number of firms in the control group and a myriad of products each one sells. Nevertheless, it is worthwhile to note many of the early contributions to the pricing-based merger retrospective studies in the U.S. and in Europe, such as Kim and Singal [15], Focarelli and Penetta [16], Ashenfelter and Hosken [17], Dobson and Piga [18], Ashenfelter, Hosken [19,20], and many others.

Yet another contribution of our paper lies in studying the issue of structural remedies versus behavioral remedies. In practice this issue has been hotly debated in the antitrust community in China, whose competition authority appears to have favored the latter over the former in the past. This is a position also favored by the European Commission (2003, Article 7(1)), which states, “Structural remedies can only be imposed either where there is no equally effective behavioral remedy or where any equally effective behavioral remedy would be more burdensome for the undertaking concerned than the structural remedy.” But Hoehn [21] shows structural remedies tend to dominate in his study of 234 merger remedies decisions in six EU countries and Switzerland. In the United States, Kwoka and Moss [22] reported that the 2011 Antitrust Division Policy Guide to Merger Remedies offered a more favorable view of behavioral remedies relative to structural remedies than the past U.S. Department of Justice policies and practices. What we found in China is that both types of remedies tend to be effective, but their effectiveness unfolds in different ways in that structural remedies usually cause an abrupt, severe impact immediately after the merger decision, while the effect of behavioral remedies kicks in gradually over time. In the end both remedies would achieve the same desired result, suggesting that the debate over this issue seems to be a moot point after all.

Relevant to our paper, we also need to cite the literature of studies of merger controls in China, starting with Lin and Zhao [23] who provided a comprehensive introduction of the merger control process in China. Noticeably they found no clear evidence of industrial policy considerations in the eight published decisions as of their writing. Choi and Youn [24] discussed the fundamental differences between the objectives of Chinese competition law and the objectives of competition law in Western countries, and explained how this eventually results in dissimilar decision outcomes. Using the revealed preference approach, Shan, Tan [25] investigated the welfare standard that the Anti-monopoly Law in China seeks to maximize by examining both its stated language and seven merger control actions taken by the competition authority. Han and Ryan [26] provided a legal analysis of remedies in Chinese merger control law with several case studies. One commonality among this strand of literature is that they are all qualitative studies, while ours is the first attempt at understanding the remedy issue in China in a more rigorous and quantitative manner.

The rest of the paper is organized as follows. The next section introduces our method and the data regarding 8 merger cases at issue. Section 3 presents the results of our analysis. Section 4 concludes with a brief discussion of our overall assessment of the Chinese competition authority’s merger review performance.

## 2. Method and data

In this section, we first describe our method, called the synthetic control method (SCM), to create a counterfactual of a merging entity involving remedies based on the data from those mergers in the same industry that are cleared by the competition authority unconditionally. We then discuss the data we use for this exercise.

The SCM method, first proposed by Abadie and Gardeazabal [5] and later assisted with a software package as explained in Abadie, Diamond [27] and Galiani and Quistorff [28], is premised upon the idea of identifying the optimal weighted average vector, in the sense of closely fitting the state of the merger entity of interest prior to the intervention by minimizing the Mahalanobis distance. The identified optimal weighted average vector can then be used to linearly combine the relevant variables for entities in the control group, or the set of those mergers approved unconditionally in our case, to project the likely scenario that would have happened without remedies to a merger entity with remedies, or counterfactual as it is called in the literature. This method has some advantages over the traditional difference-in-difference (DID) method that typically gives all untreated units the same weight.

Mathematically, suppose there are J+1 firms, with J representing the number of firms involved in merger cases approved unconditionally, and 1 representing the merger firm approved with policy intervention in the form of some kind of remedies. Let $T_{\text{0}}$ denote the time when the policy intervention is implemented. All firms are unaffected by any policy in the period, $\left[\text{1},T_{\text{0}}\right)$; other than for 1 for the period, $\left[T_{\text{0}},T\right]$. We use $Y_{jt}^{N}$ to denote the market power of firm j without policy intervention at time t, for firms j=1, 2, ...J+1. For firm 1, $Y_{\text{1}t}^{N}$ is essentially the counterfactual we are creating as of $T_{\text{0}}$. $Y_{jt}^{I}$ denotes the observed market power of the firm j at time *t*, $t\in [T_{\text{0}},T]$, when the policy intervention is implemented, if it is implemented. Then, $\alpha_{\text{1}t}=Y_{\text{1}t}^{I}-Y_{\text{1}t}^{N}$, $t\in [T_{\text{0}},T]$, shows the difference in market power brought about by the policy intervention to firm 1 at time t. We use the factor model proposed by Abadie, Diamond [8] to estimate:


$$Y_{jt}^{N}=\delta_{t}+\theta_{t}Z_{j}+\lambda_{t}u_{j}+\varepsilon_{jt}$$ (1)

where $\delta_{t}$ denotes a time fixed effect; $Z_{j}$ is a (r×1) vector of observed covariates, which are not affected by the policy intervention, and $\theta_{t}$ is a (1×r) vector of their parameters at time *t*. Let $u_{j}$ denotes a (q×1) vector of variables reflecting unobservable individual fixed effects, and $\lambda_{t}$ is a (1×q) vector of common factors. $\varepsilon_{jt}$ represents zero mean individual transitory shocks. Now consider a vector of weights $\text{W}=({\text{w}}_{\text{2}},{\text{w}}_{\text{3}}...{\text{w}}_{\text{J}+\text{1}})$,${\text{ w}}_{\text{j}}\geq \text{0}$, j=2, 3...J+1and $\sum_{\text{j}=\text{2}}^{\text{J}+\text{1}}{\text{w}}_{\text{j}}=\text{1}$. Then equation (1) can be rewritten as the following for firm 1:


$${\hat{Y}}_{\text{1}t}^{N}=\sum {\mathrm{\backslash nolimits}}_{j=\text{2}}^{J+\text{1}}w_{j}Y_{jt}^{N}=\delta_{t}+\theta_{t}\sum {\mathrm{\backslash nolimits}}_{j=\text{2}}^{J+\text{1}}w_{j}Z_{j}+\lambda_{t}\sum {\mathrm{\backslash nolimits}}_{j=\text{2}}^{J+\text{1}}w_{j}u_{j}+\sum {\mathrm{\backslash nolimits}}_{j=\text{2}}^{J+\text{1}}w_{j}\varepsilon_{jt}$$ (2)

Abadie, Diamond [8] proved that an optimal set of weights, $W^{*}=(w_{\text{2}}^{*},w_{\text{3}}^{*}...w_{J+\text{1}}^{*})$, can be approximated asymptotically for equation (2) by minimizing the Mahalanobis distance under certain regularity conditions. Then the estimator of the effect of policy intervention, that is the difference between the counterfactual and the observed market power, ${\hat{\alpha }}_{\text{1}t}$, can be calculated as follows:


$${\hat{\alpha }}_{\text{1}t}=Y_{\text{1}t}^{I}-{\hat{Y}}_{\text{1}t}^{N}=Y_{\text{1}t}^{I}-\sum {\mathrm{\backslash nolimits}}_{j=\text{2}}^{J+\text{1}}w_{j}^{*}Y_{jt}^{N}$$ (3)

${\hat{\alpha }}_{\text{1}t}$is one of the most important variables to evaluate the impact of remedies, as its interpretation would be measuring the difference between the observed competition trajectory under remedies and the hypothetical trajectory that would have resulted in the absence of remedies. To address the other benchmark comparison with the average pre-merger competition level, we measure the difference between the observed competition trajectory under remedies and the hypothetical average pre-merger level of competition, ${\hat{\beta }}_{\text{1}t}$, that can be calculated as follows:


$${\hat{\beta }}_{\text{1}t}=Y_{\text{1}t}^{I}-\frac{\sum_{t=\text{1}}^{T_{\text{0}}-\text{1}}{\hat{Y}}_{\text{1}t}^{N}}{\sum_{t=\text{1}}^{T_{\text{0}}-\text{1}}t}=Y_{\text{1}t}^{I}-\frac{\sum_{t=\text{1}}^{T_{\text{0}}-\text{1}}\sum_{j=\text{2}}^{J+\text{1}}w_{j}^{*}Y_{jt}^{N}}{\sum_{t=\text{1}}^{T_{\text{0}}-\text{1}}t}$$ (4)

The data used in this paper comes from several sources. Since the SCM method is based on building a counterfactual for a merger entity at issue with a weighting algorithm in a factor model to determine the combination of the control units’ data from a control group of firms of a similar situation, our first step is to establish matches between restrictive mergers and those unrestrictive mergers of a similar situation, meaning those cases that have passed the merger review process unconditionally but involving firms that are also similar to the merger entity at issue that has passed the merger review process conditionally with remedies. Altogether there are 39 merger decisions cleared with restrictive conditions and 2,396 merger decisions cleared without remedies in the period from 2008 to 2018. Out of the 39 cases, 7 cases are excluded, as the remedies have changed or terminated because of various reasons. Let’s call the remaining cases as belonging to group A.

Out of the 2,396 unconditional cases, we exclude 517 cases that do not contain information about merger decision dates by the competition authority. Let’s call the remaining cases as belonging to group B. The reason why we exclude these cases is because of the event date requirement that the SCM method critically depends on building a counterfactual. Following Duso, Gugler [29], who use the merger decision date as the event date, we choose the quarter of the year in which the announcement date of the merger decision happens as the event date, denoted by $T_{\text{0}}$ in this paper, where $T_{\text{0}}=\text{0}$. For the pre-treatment period, we utilize a 5-year (20-quarter) horizon prior to the merger event. This duration is commonly employed in the synthetic control literature and allows for sufficient data to construct a reliable counterfactual, as confirmed by our sensitivity tests (see In-time placebo test). To assess remedy success, we rely on the criterion established by the Federal Trade Commission [30], which considers a remedy successful if competition in the relevant market either remains at or returns to its pre-merger level within 2–3 years post-merger. Accordingly, our analysis focuses on a 2-year post-event evaluation window to directly test the policy effect within the FTC-specified time frame for recovery.

The next step is to identify matches of companies in group A and group B in that each match needs to belong to the same industry based on the 3-digit industry code. We choose the 3-digit industry code instead of the 4-digit industry code, because the 4-digit industry code would generate too small a sample for meaningful analysis across our entire selected sample. Gugler, Mueller [14] even used the 2-digit industry code in his study. Nevertheless, we still include a robustness test based on the 4-digit industry code for a subset of cases where there is an adequate sample size in the control group. We also take into consideration the availability of public financial data and other requirements of the SCM method. The data screening exercise as described so far then results in 8 firms in 8 cases from group A, and for each one of these cases, 16–24 cases/firms were identified from group B, covering altogether 6 industries and about 150 companies, including pharmaceuticals, healthcare equipment and supplies, automobiles, electrical equipment, chemicals, and semi-conductor products and equipment. Table 1 summarizes the information about these 8 cases and their matches.

**Table 1:** Classification of sample by remedies type.

Case (treatment)	Publication Date	Merger Type	Industry	Firms (treatment units)	Number of control units
**1. Structural Remedies**					
Pfizer/Wyeth	2009.9.29	Acquisition	Pharmaceuticals	Pfizer	16
NXP/Freescale	2015.11.27	Acquisition	Semi-conductor Products and Equipment	NXP Semiconductors	18
**2. Behavioral Remedies**					
Broadcom/ Brocade Communications	2017.8.22	Acquisition	Semi-conductor Products and Equipment	Broadcom	17
Advanced Semiconductor/ Siliconware Precision	2017.11.24	Acquisition	Semi-conductor Products and Equipment	Advanced Semiconductor (ASX)	18
Novartis/Alcon	2010.8.13	Acquisition	Pharmaceuticals/ Healthcare Equipment and Supplies	Novartis	17
Corun/Toyota China/ PEVE/Xin Zhongyuan/ Toyota Tsusho	2014.7.2	Joint Venture	Automobiles/ Electrical Equipment	Corun	24
**3. Hybrid Remedies**					
Dow Chemical/Dupont	2017.5.2	Merger	Chemicals	Dupont	23
Linde/Praxair	2018.9.30	Merger	Chemicals	Praxair	23

Once the firm list is identified, we then need to identify a set of variables for analysis. Based on the SCM method, we need data for two sets of variables, the outcome variable and the matching variables as they are usually called in the SCM framework. Our outcome variable is to measure a firm’s market power, which we use the price-cost margin (PCM) as a proxy, calculated based on the sales revenue and the cost of goods sold of a firm. In many economic theories, competition is related to the relative size of a mark-up on the cost price as a component of the output price, but data on the price-cost margin (PCM) are generally not available in the financial markets [31]. As already explained in the introduction section, like other classical literature, we use gross margin as a proxy variable for PCM to measure competition.

The purpose of the matching variables in the SCM framework is not for hypothesis testing, so the variables’ direction of effect on the outcome variable is not important. Instead, their function is strictly to improve the pre-treatment fit. In general, financial analyses focus on four main aspects of a firm’s operational performance, including profitability indicators, solvency indicators, development ability indicators and operational capacity indicators. Following this line of thinking, we arrive at a set of outcome and matching variables illustrated in Table 2.

**Table 2:** Variable definitions.

Outcome and matching variables	
Variable name	Definition
Price-cost margin	Sales revenue minus cost of goods sold and divided by sales revenue
Size	Natural log of total assets
Ratio of liabilities to assets	Total debts divided by total assets
Rate of return on total assets	EBIT divided by average total assets
Total asset turnover ratio	Operating income divided by average total assets
Cash flow ratio	Generating from operating activities net cash flow divided by total assets
Quarter-on-quarter GDP growth	${GDP}_{t}-{GDP}_{t-\text{1}}{/GDP}_{t-\text{1}}$
Merger type	Dummy is 1 when type of concentration is Merger, 2 when type is Acquisition, and 3 others
Stock type	Dummy is 1 when type of stock is Chinese stock, 2 when type is U.S. stock, and 3 others
Industry type^a^	Binary variable
Pre-treatment outcome variable values^b^	$Y_{j-\text{1}}^{N}$, $Y_{j-\text{5}}^{N}$, $Y_{j-\text{1}\text{0}}^{N}$, $Y_{j-\text{1}\text{5}}^{N}$

^a^If a merger affects more than one industry, we pool the firms in all industries concerned. Among them, 2 cases of concentration involve two industries, while the other cases involve only one industry. This can be found in Table 1.

^b^The minus subscript represents the number of quarters prior to the event date, which is the quarter in which the merger decision is approved.

We use four types of matching variables: (i) firm characteristics variables: size, ratio of liabilities to assets, rate of return on total assets, total asset turnover ratio and cash flow ratio; (ii) national macro variables: quarter-on-quarter GDP growth at constant prices; (iii) dummy variables: merger type, stock type and industry type variables; and (iv) the outcome variable for the pre-intervention period, specifically choosing pre-intervention values of the outcome variable, this is because they play a crucial role in reproducing the unobserved factor loading$\;{\text{ u}}_{\text{j}}$ in the factor model [32]. The financial data source is the Wind database. And the quarterly GDP data of the countries involved are from the OECD. Finally, to avoid the effect of extreme data points for some firms in certain years, we apply a Winsorized tailing treatment of 1% before and after for all continuous variables in the sample.

## 3. Results

We present results regarding the effect of different remedies on the concentration of undertakings in this section, followed by four additional tests to evaluate the robustness of the estimated effect.

### 3.1. Effect of remedies

**Fig 1:**
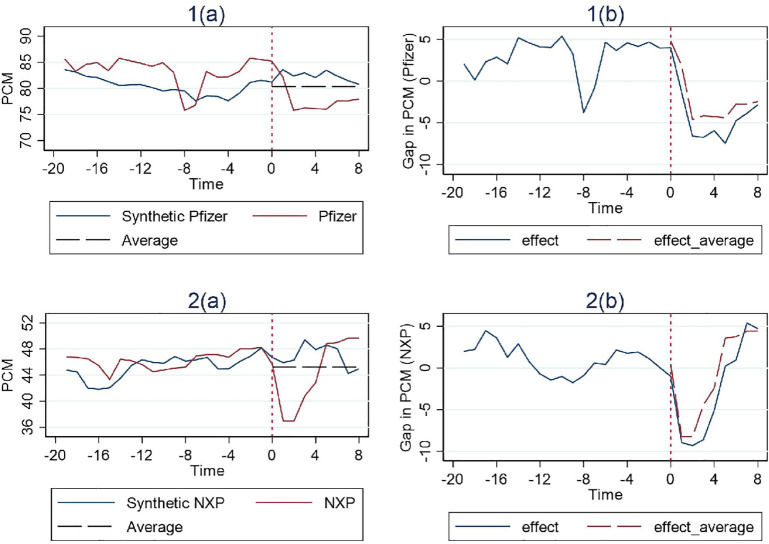
Results for cases with structural remedies (Simple Average).

**Fig 2:**
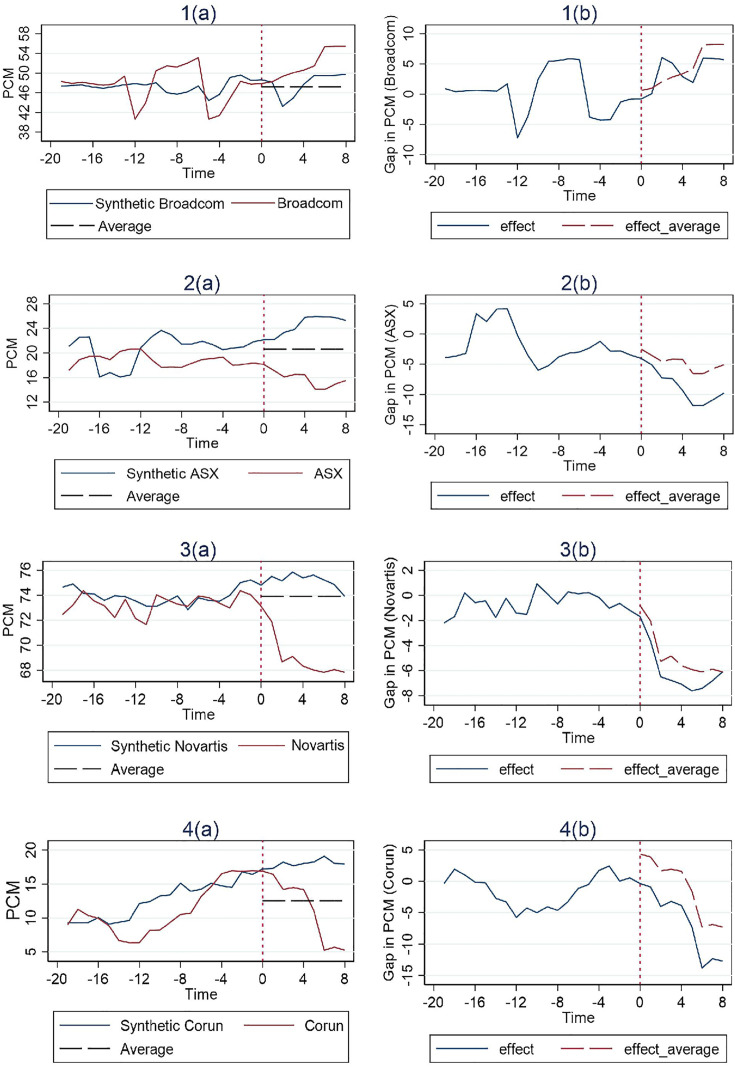
Results for cases with behavioral remedies (Simple Average).

**Fig 3:**
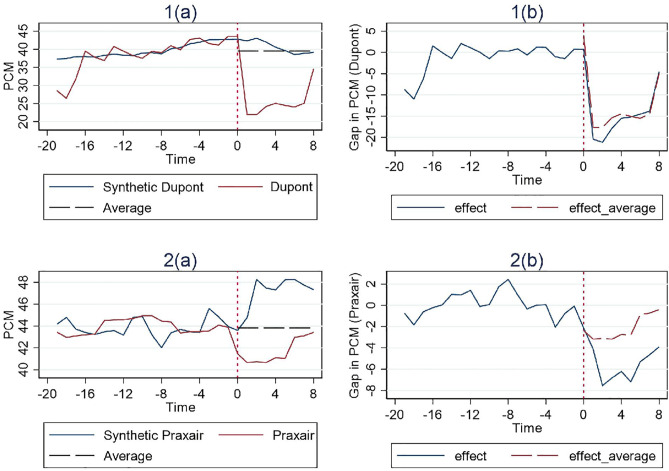
Results for cases with hybrid remedies (Simple Average).

In Fig 1, 2 and 3, the vertical dashed line corresponding to time 0 represents the event date. The two solid lines represent the PCM of the merger entity at issue and the associated control group’s synthetic PCM construct based on the SCM method. To the right of the event date, a horizontal dashed line represents the average pre-merger PCM level of the control group. The actual merger entity’s post-merger PCM level can be compared to both the synthetic post-merger PCM construct and the average pre-merger PCM level of the control group. If the merger entity’s post-merger PCM level is lower than both, we then have a clean case of merger review success. If however it is higher than the average pre-merger PCM level of the control group but lower than their synthetic post-merger PCM construct, then a definitive conclusion of merger review success cannot be drawn, since the post-merger PCM construct could possibly be the result of a merger review failure on the part of the competition authority in the first place, or possibly, something it intentionally tolerates.

The trends of the PCM for each treatment unit and its corresponding synthetic control unit are illustrated in the figures on the left, while the differences between them are depicted in the figure on the right. And the effect of the remedies is estimated by examining whether a considerable difference exists between the trajectory of the measure for the treatment unit and its synthetic control after the event date. Additionally, the pre-treatment match quality can be inferred from its deviation between the synthetic control and the treatment unit during the pre-intervention period.

We have 2 cases involving structural remedies as illustrated in Fig 1, Pfizer and NXP. The NXP case shows a particularly much better fit by the respective synthetic control prior to the event date, with the deviation basically fluctuating around 0. This observation indicates that the synthetic trajectory of PCM in the post-intervention period should provide an accurate counterfactual representation of the trajectory that would have occurred if NXP had not been hit with structural remedies after the merger. Nonetheless, examination of the gap between the factual and synthetic trajectories in the post-intervention period as shown in figures on the right for both cases, reveals a clearly negative estimated effect of the structural remedies. This effect appears to be large and immediate within the first four quarters post-merger. Over time, however, the gap diminishes gradually in subsequent quarters.

Furthermore, Fig 1 illustrates that the post-merger competition level in both cases compare favorably against the average competition level of the control group prior to the merger. Given that both comparative assessments are satisfied, it is reasonable to conclude that the structural remedies implemented in these two cases do serve their purpose by reducing PCM and thereby enhancing competition immediately after the merger. However, this desired effect appears to be transient, as PCM gradually crawls back to the level comparable to the control group with no remedies after only four quarters.

We have 4 cases involving behavioral remedies. Among these, three cases—excluding Broadcom—show a consistent pattern of a significant negative effect on PCM for both comparison tests, as illustrated in Fig 2. Notably, this adverse effect unfolds gradually over time. This contrasts with cases involving structural remedies, where the negative effect on PCM manifests immediately within the first year after the policy intervention. Such a difference is anticipated, given that the nature of structural remedies that usually demand major organizational changes typically involving divesting assets. Within our data time period, PCM in all cases eventually did show a modest recovery later, other than that the Broadcom case appears to be an anomaly in that PCM actually trended upwards, indicating an opposite effect to what the competition authority would have desired of.

Finally, Fig 3 illustrates the results for the 2 cases with hybrid remedies, both of which indicate effectiveness as evidenced by passing both comparison tests. And these results align more closly with those observed in structural remedy cases, characterized by an immediate and pronounced decline in PCM after the event date, followed by steady recovery over time.

### 3.2. Robustness tests

#### 3.2.1. Robustness test with a different averaging method.

In the previous section we construct the synthetic pre-merger PCM level of the control group by just taking the simple average before the event date. It stands to argue that heavier weighting should be placed on more recent data. So here we conduct a robustness test based on a weighted average method that assigns heavier weighting to more current data points.

Suppose the synthetic PCM level of the control group in time t, $t\in [{\text{1},T}_{\text{0}})$ is ${PCM}_{t}$. Then the weight of ${PCM}_{t}$ is $t/\frac{T_{\text{0}}(T_{\text{0}}-\text{1})}{\text{2}}$, and the average pre-merger PCM will become $\sum {\mathrm{\backslash nolimits}}_{t=\text{1}}^{T_{\text{0}}-\text{1}}PCM_{t}\;\cdot \;t/\frac{T_{\text{0}}(T_{\text{0}}-\text{1})}{\text{2}}$.

**Fig 4:**
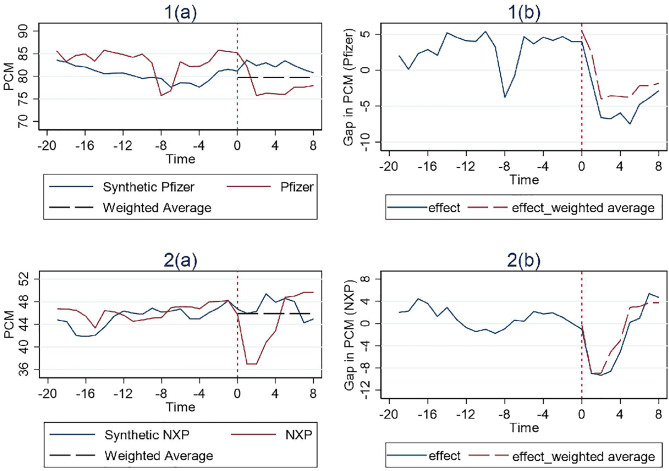
Results for cases with structural remedies (Weighted Average).

**Fig 5:**
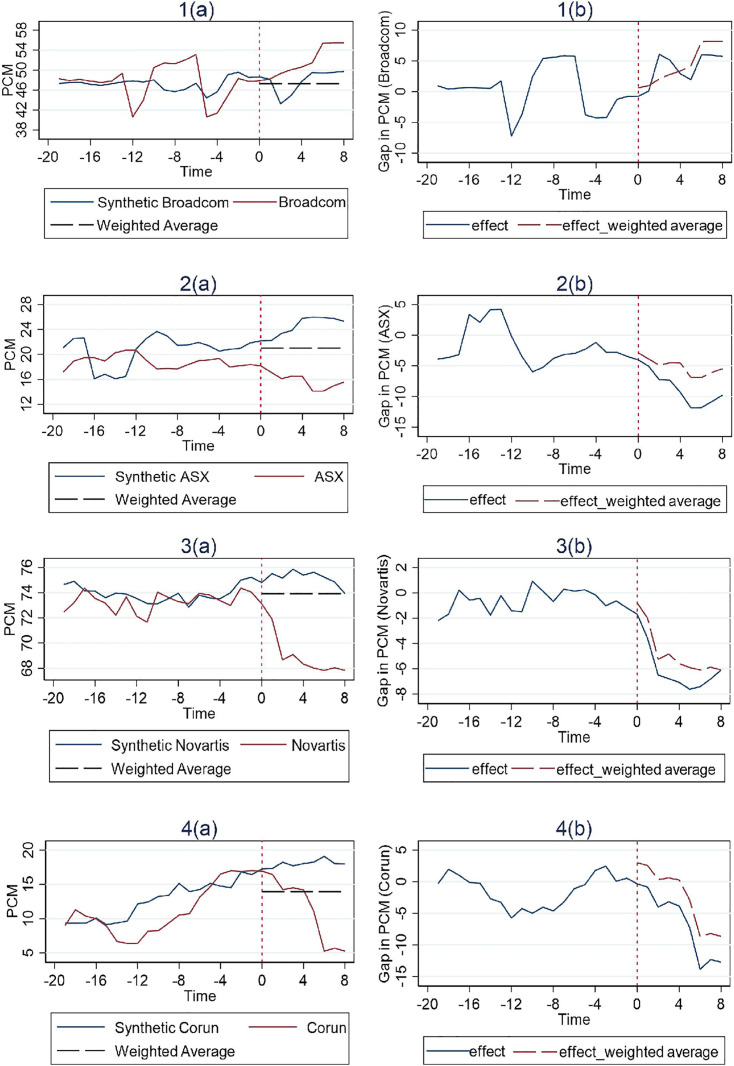
Results for cases with behavioral remedies (Weighted Average).

**Fig 6:**
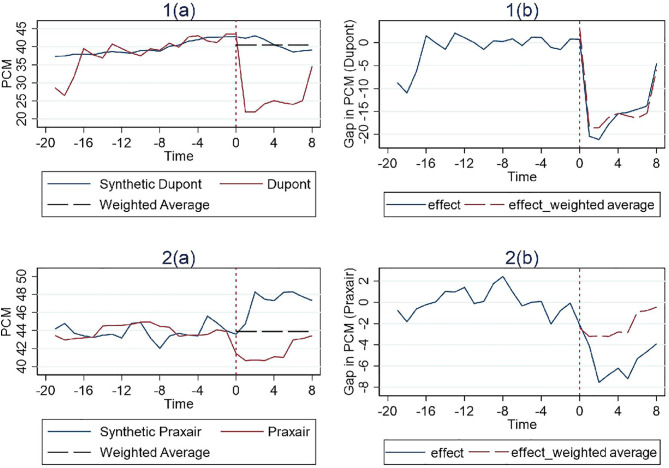
Results for cases with hybrid remedies (Weighted Average).

The results in Fig 4–6 indicate that there is little difference between using the simple average and weighted average methods in seven cases that show remedy effectiveness other than the Broadcom case. From both the perspective of comparing with the synthetic PCM construct or the pre-merger PCM level, the results remain the same.

#### 3.2.2. In-space placebo test.

The customary robustness test in the synthetic control method is also known as the placebo test, which attempts to answer the question whether the gap between the factual and the synthetic values uncovered in the empirical analysis is truly influenced by the policy intervention, or merely a coincidence. The idea of the placebo test is to similarly use the synthetic control method to analyze the effect of the intervention on control units unaffected by the intervention, called the in-space placebo test in Abadie, Diamond [33]. Galiani and Quistorff [28] improved upon the Abadie, Diamond [33] method by calculating the p-values in the placebo tests.

Table 3 shows the result of the placebo test: p-values, and the pre- and post-treatment root-mean-square prediction error (RMSPE). Except for Pfizer and Broadcom, all firms have a treatment effect significantly different from zero, since the “P-value_joint” numbers in the table are similar to the standardized p-values being close to 0 in conventional regression analyses. What it means is that the proportion of the control units has an estimated effect at least as large as the treated units across all post-treatment periods in consideration of the pre-treatment match quality. So it is reasonable to infer that the implemented remedies have a statistically significant negative effect on PCM, aka market power, in 6 out of 8 cases, excluding Pfizer and Broadcom, regardless of comparing to our post-merger synthetic PCM construct or to the pre-merger PCM level.

**Table 3:** The result of in-space placebo tests.

	Structual remedies		Behavioral remedies				Hybrid remedies	
	(1) Pfizer	(2) NXP	(1) Broadcom	(2) ASX	(3) Novartis	(4) Corun	(1) Dupont	(2) Praxair
P-values_joint	0.875	0.000***	0.059	0.000***	0.000***	0.000***	0.000***	0.000***
pre_RMSPE	3.767	1.952	3.679	3.483	1.025	2.885	3.661	1.108
post_RMSPE	5.515	6.328	4.736	9.525	6.624	8.660	16.117	5.917

* p < 0.05, ** p < 0.01, *** p < 0.001

#### 3.2.3. In-time placebo test.

Next, for these remaining 6 cases, we conduct an additional robustness test to further assess the validity of our findings regarding the effect of remedies. Following Abadie, Diamond [33], we conduct a in-time placebo test. The idea of this test is to show that the divergence between the factual and counterfactual trajectories would only happen immediately after the actual event date, and if we hypothetically applied another event date—such as one year earlier, or at $T_{\text{0}}=-\text{4}$, as conducted in this analysis—the divergence would still happen at the original event date rather than at the placebo date $T_{\text{0}}=-\text{4}$. Given that data are available for the twenty quarters prior to the event date, this approach enables us to evaluate whether the method produces large estimated effects when applied to dates before the intervention.

**Fig 7:**
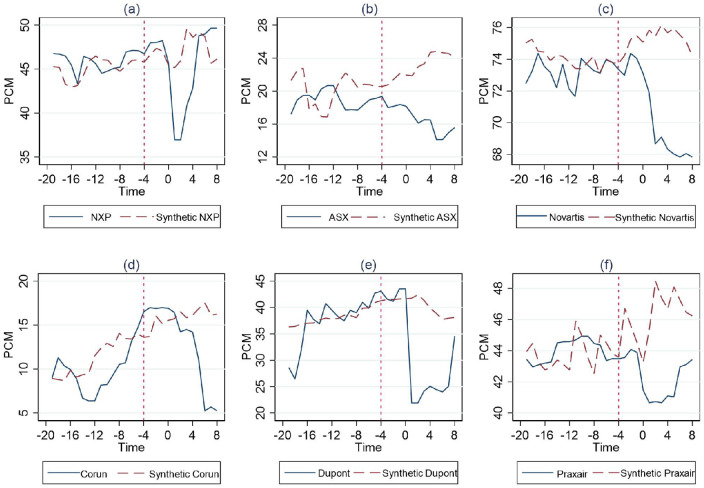
The result of in-time placebo test.

This test shows the divergence indeed happens starting from the event date. We can then arrive at two important robustness test conclusions. First, before the start of the actual intervention, synthetic units consistently achieve strong pre-treatment fit across all reassigned cases confirming model reliability despite the constrained pretreatment window. Second, significant divergences between placebo paths and observed outcomes emerge only at the time of actual intervention, not before, and closely mirror the gap dynamics of true treated units (Fig 1–3 vs. Fig 7). These patterns confirm that our estimator correctly tracks trajectories pre-intervention while identifying effect onset with temporal precision. The absence of “phantom effects” predating actual interventions and the specificity of divergence timing jointly reinforce that pretreatment fit suffices and estimated structural breaks reflect genuine causal effects of the remedies, not model artifacts.

#### 3.2.4. Robustness test with the 4-digit industry code.

**Fig 8:**
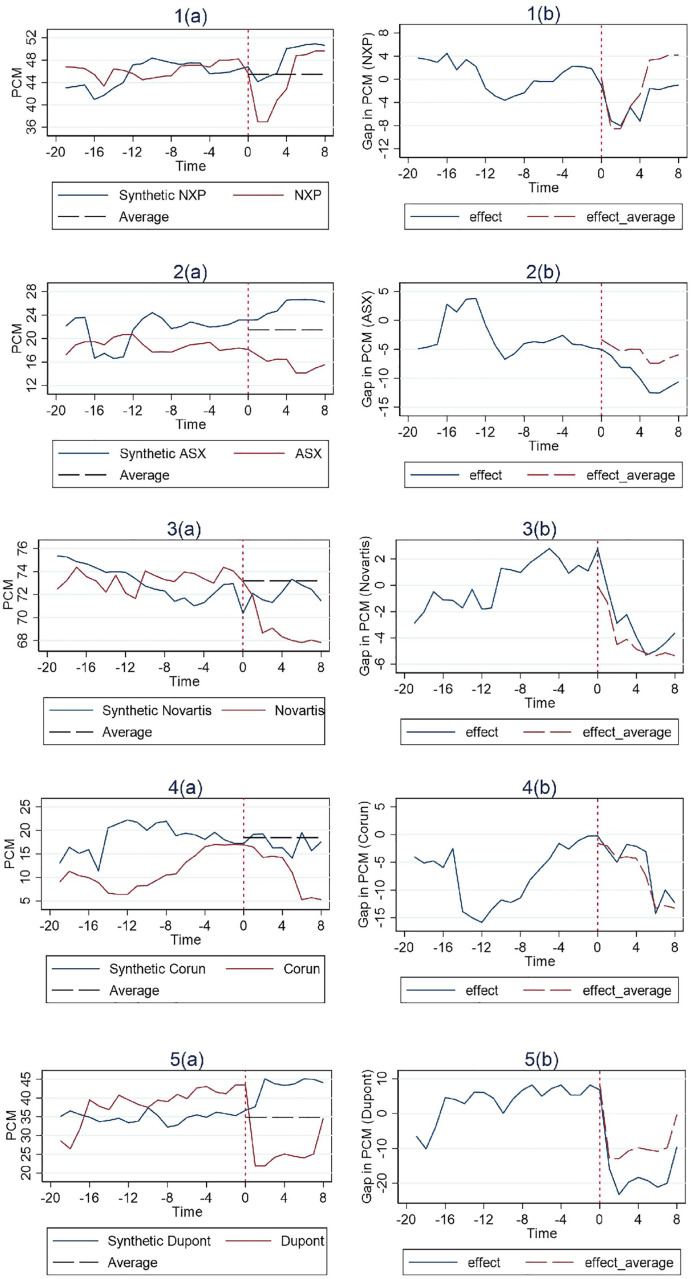
The result using the 4-digit industry code.

In this robustness test, we examine the impact of using data based on the 4-digit industry code. The advantage of going one level down in detail in industry code is obvious as the control group would resemble more the merger entity at issue, and thus theoretically improving the counterfactual construction under the SCM. However, this increased specificity comes with the drawback of a reduced sample size. Indeed we have to exclude one case (Praxair) in which there is only one firm remaining in the control group. Additionally, the Pfizer case is omitted because it exhibits poor performance in the in-space placebo test as shown in the previous subsection. The Broadcom case is also excluded, given its consistently poor performance throughout our analysis.. All of the five remaining cases’ performance demonstrates robustness satisfactorily, since there is no noticeable difference from our base model as illustrated in Fig 8.

## 4. Conclusions

This paper uses a new method called the synthetic control method (SCM) to evaluate merger control effectiveness by creating a counterfactual of a merging entity involving remedies based on the data from those mergers in the same industry that are cleared by the competition authority without remedies. Specifically, we compare the actual post-merger PCM level of merger entity with: 1). the average pre-merger PCM level of the control group; 2). the synthetic post-merger PCM construct of the control group. One of the rationales behind this approach is to incorporate the competition authority’s possible expectation with respect to the post-merger competition level. We use data concerning 8 merger cases cleared by the competition authority with remedies, and about 150 cases cleared without remedies. Our interest is to investigate whether these remedies are effective, and whether different types of remedies would result in different effects.

Our analysis shows that, overall, merger remedies are indeed effective in mitigating the anti-competitive effect in terms of decreasing the PCM, which serves as a proxy for assessing the degree of market competition. Out of the 8 cases under investigation, all but one sees the PCM value immediately declining after the merger.

However, the effect of remedies varies according to their type. The structural remedy demonstrate a capacity to significantly and rapidly reduce the PCM, thereby effectively curbing the anti-competitive effect. In contrast, the behavioral remedy appears to be working out its way gradually over time in decreasing the PCM, and its magnitude of decline also appears to be modest. This gradual effect is consistent with theoretical expectations, as behavioral remedies typically require time to manifest their intended outcomes.

Another interesting phenomenon we observe is that the PCM did start to trend back after some time, and particularly the structural remedy cases trending back more quickly and with a larger magnitude. This is probably because typically behavioral remedies in China would invoke a government process for the competition authority to keep monitoring the firm for compliance with the remedies for some time, which usually can last up to 3–5 years.

Our analysis poses an interesting question. Even though remedies do appear to be effective immediately after the competition authority’s decision, how long lasting they are and whether they are able to maintain the same level of competition prior to the merger remain to be unanswered questions, perhaps worthy of future research.

## Supporting information

S1 Data (ZIP)

## References

[1] Nie M. The trend of monopoly in merging with foreign companies and the improvement on related legal system. Social Science. 2005;09:36–43.

[2] Huang X. Research on the Relationship between WTO, Economic Globalization, Knowledge Economy, and China’s Anti-Monopoly Legislation. Political-Legal Forum. 2001;(05):13–21. https://kns.cnki.net/kcms/detail/detail.aspx?dbname=cjfd2001&filename=ZFLT200105001&dbcode=cjfd

[3] Owen BM, Sun S, Zheng W. Antitrust in China: The Problem of Incentive Compatibility. Journal of Competition Law & Economics. 2005;1(1):123–48. doi: 10.1093/joclec/nhi004

[4] Carlton DW. Why we need to measure the effect of merger policy and how to do it. National Bureau of Economic Research. 2009.

[5] Abadie A, Gardeazabal J. The Economic Costs of Conflict: A Case Study of the Basque Country. American Economic Review. 2003;93(1):113–32. doi: 10.1257/000282803321455188

[6] Goin DE, Rudolph KE, Ahern J. Impact of drought on crime in California: A synthetic control approach. PLoS One. 2017;12(10):e0185629. doi: 10.1371/journal.pone.0185629 28977002 PMC5627925

[7] Mills MC, Rüttenauer T. The effect of mandatory COVID-19 certificates on vaccine uptake: synthetic-control modelling of six countries. Lancet Public Health. 2022;7(1):e15–22. doi: 10.1016/S2468-2667(21)00273-5 34914925 PMC8668192

[8] Abadie A, Diamond A, Hainmueller J. Synthetic Control Methods for Comparative Case Studies: Estimating the Effect of California’s Tobacco Control Program. Journal of the American Statistical Association. 2010;105(490):493–505. doi: 10.1198/jasa.2009.ap08746

[9] Gietel-Basten S, Han X, Cheng Y. Assessing the impact of the “one-child policy” in China: A synthetic control approach. PLoS One. 2019;14(11):e0220170. doi: 10.1371/journal.pone.0220170 31693666 PMC6834373

[10] Chen X, Lin B. Towards carbon neutrality by implementing carbon emissions trading scheme: Policy evaluation in China. Energy Policy. 2021;157:112510. doi: 10.1016/j.enpol.2021.112510

[11] Gugler K, Szücs F. Merger externalities in oligopolistic markets. International Journal of Industrial Organization. 2016;47:230–54. doi: 10.1016/j.ijindorg.2016.05.003

[12] Mehta A, Miller NH. Choosing the Appropriate Control Group in Merger Evaluation. More Pros and Cons of Merger Control. 2012:189.

[13] De Loecker J, Eeckhout J, Unger G. The Rise of Market Power and the Macroeconomic Implications*. The Quarterly Journal of Economics. 2020;135(2):561–644. doi: 10.1093/qje/qjz041

[14] Gugler K, Mueller DC, Yurtoglu BB, Zulehner C. The effects of mergers: an international comparison. International Journal of Industrial Organization. 2003;21(5):625–53. doi: 10.1016/s0167-7187(02)00107-8

[15] Kim EH, Singal V. Mergers and market power: Evidence from the airline industry. The American Economic Review. 1993;83(3):549–69.

[16] Focarelli D, Panetta F. Are Mergers Beneficial to Consumers? Evidence from the Market for Bank Deposits. American Economic Review. 2003;93(4):1152–72. doi: 10.1257/000282803769206241

[17] Ashenfelter O, Hosken D. The Effect of Mergers on Consumer Prices: Evidence from Five Mergers on the Enforcement Margin. The Journal of Law and Economics. 2010;53(3):417–66. doi: 10.1086/605092

[18] Dobson PW, Piga CA. The Impact Of Mergers On Fares Structure: Evidence From European Low‐cost Airlines. Economic Inquiry. 2011;51(2):1196–217. doi: 10.1111/j.1465-7295.2011.00392.x

[19] Ashenfelter OC, Hosken DS, Weinberg MC. Corrigendum: The Price Effects of a Large Merger of Manufacturers: A Case Study of Maytag Whirlpool. American Economic Journal: Economic Policy. 2014;6(1):308–9. doi: 10.1257/pol.6.1.308

[20] Ashenfelter OC, Hosken DS, Weinberg MC. Efficiencies brewed: pricing and consolidation in the US beer industry. The RAND J of Economics. 2015;46(2):328–61. doi: 10.1111/1756-2171.12092

[21] Hoehn T. Structure Versus Conduct – A Comparison of the National Merger Remedies Practice in Seven European Countries. International Journal of the Economics of Business. 2010;17(1):9–32. doi: 10.1080/13571510903516938

[22] Kwoka JE, Moss DL. Behavioral Merger Remedies: Evaluation and Implications for Antitrust Enforcement. The Antitrust Bulletin. 2012;57(4):979–1011. doi: 10.1177/0003603x1205700410

[23] Lin P, Zhao J. Merger Control Policy Under China’s Anti-Monopoly Law. Rev Ind Organ. 2012;41(1–2):109–32. doi: 10.1007/s11151-012-9345-9

[24] Choi YS, Youn SY. The Enforcement of Merger Control in China: A Critical Analysis of Current Decisions by MOFCOM. IIC. 2013;44(8):948–72. doi: 10.1007/s40319-013-0128-0

[25] Shan P, Tan G, Wilkie SJ, Williams MA. China’s Anti-Monopoly Law: What is the Welfare Standard?. Rev Ind Organ. 2012;41(1–2):31–52. doi: 10.1007/s11151-012-9349-5

[26] Han M, Ryan S. Remedies in Chinese merger control law-themes, recent cases and developments. Business Law International. 2014;15(3):201–22.

[27] Abadie A, Diamond A, Hainmueller J. Synth: AnRPackage for Synthetic Control Methods in Comparative Case Studies. J Stat Soft. 2011;42(13). doi: 10.18637/jss.v042.i13

[28] Galiani S, Quistorff B. The Synth_Runner Package: Utilities to Automate Synthetic Control Estimation Using Synth. The Stata Journal. 2017;17(4):834–49. doi: 10.1177/1536867x1701700404

[29] Duso T, Gugler K, Yurtoglu BB. How effective is European merger control?. European Economic Review. 2011;55(7):980–1006. doi: 10.1016/j.euroecorev.2011.04.003

[30] FTC. The FTC’s merger remedies 2006-2012. Federal Trade Commission. 2017. https://www.ftc.gov/system/files/documents/reports/ftcs-merger-remedies-2006-2012-report-bureaus-competition-economics/p143100_ftc_merger_remedies_2006-2012.pdf

[31] Bikker JA, Spierdijk L. Measuring and explaining competition in the financial sector. Journal of Applied Business & Economics. 2010;11(1):11–42.

[32] Abadie A. Using Synthetic Controls: Feasibility, Data Requirements, and Methodological Aspects. Journal of Economic Literature. 2021;59(2):391–425. doi: 10.1257/jel.20191450

[33] Abadie A, Diamond A, Hainmueller J. Comparative Politics and the Synthetic Control Method. American Journal of Political Science. 2014;59(2):495–510. doi: 10.1111/ajps.12116

